# Post-exercise Hot Water Immersion Elicits Heat Acclimation Adaptations in Endurance Trained and Recreationally Active Individuals

**DOI:** 10.3389/fphys.2018.01824

**Published:** 2018-12-18

**Authors:** Michael J. Zurawlew, Jessica A. Mee, Neil P. Walsh

**Affiliations:** Extremes Research Group, College of Human Sciences, Bangor University, Bangor, United Kingdom

**Keywords:** heat, acclimation, hot water, thermal strain, training, running

## Abstract

Hot water immersion (HWI) after exercise on 6 consecutive days in temperate conditions has been shown to provide heat acclimation adaptations in a recreationally active population. Endurance athletes experience frequent, sustained elevations in body temperature during training and competition; as a consequence, endurance athletes are considered to be partially heat acclimatized. It is therefore important to understand the extent to which endurance trained individuals may benefit from heat acclimation by post-exercise HWI. To this end, we compared the responses of eight endurance trained and eight recreationally active males (habitual weekly endurance exercise: 9 h vs. 3 h) to a 6-day intervention involving a daily treadmill run for 40 min (65% 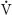
O_2max_) in temperate conditions followed immediately by HWI (≤40 min, 40°C). Before (PRE) and after the intervention (POST), hallmark heat acclimation adaptations were assessed during a 40-min treadmill run at 65% 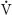
O_2max_ in the heat (33°C, 40% RH). The 6 day, post-exercise HWI intervention induced heat acclimation adaptations in both endurance trained and recreationally active individuals. Training status did not significantly influence the magnitude of heat acclimation adaptations from PRE to POST (interactions *P* > 0.05) for: the reduction in end-exercise rectal core temperature (*T*_re_, mean, endurance trained -0.36°C; recreationally active -0.47°C); the reduction in resting *T*_re_ (endurance trained -0.17°C; recreationally active -0.23°C); the reduction in *T*_re_ at sweating onset (endurance trained -0.22°C; recreationally active -0.23°C); and, the reduction in mean skin temperature (endurance trained -0.67°C; recreationally active -0.75°C: PRE to POST *P* < 0.01). Furthermore, training status did not significantly influence the observed reductions in mean 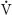
O_2_, mean metabolic energy expenditure, end-exercise physiological strain index, perceived exertion or thermal sensation (PRE to POST *P* < 0.05). Only end-exercise heart rate was influenced by training status (*P* < 0.01, interaction); whereby, recreationally active but not endurance trained individuals experienced a significant reduction in end-exercise heart rate from PRE to POST (*P* < 0.01). In summary, these findings demonstrate that post-exercise HWI presents a practical strategy to reduce thermal strain during exercise-heat-stress in endurance trained and recreationally active individuals.

## Introduction

Exercise in the heat increases physiological strain, attenuates exercise capabilities and increases susceptibility to exertional heat illness and the potentially fatal, exertional heat stroke (Young et al., 1985; Binkley et al., 2002; Racinais et al., 2015). In the early twentieth century, pioneering research on fatal heat stroke in the South African gold mines highlighted a high mortality in the first four shifts worked by miners under high heat exposure; particularly in those native to cold dry areas (Cluver, 1932; Dreosti, 1935). To mitigate the risk of heat stroke, miners acclimatized by gradual introduction to the unfavorable working conditions underground (Cluver, 1932). Current recommendations are for athletes, military personnel and others in occupations involving high heat exposure to complete a period of heat acclimation prior to competing or operating in the heat. Heat acclimation typically involves exercising in the heat on 5–14 occasions for >60-min, where core body temperature and skin temperature are elevated and perfuse sweating is initiated (Taylor, 2014; Periard et al., 2015). The adaptive responses to exercise-heat-acclimation include, but are not limited to: an earlier onset of cutaneous vasodilatation and sweating; an increase in sweating rate; a reduction in resting and exercising core body temperature; a reduction in cardiovascular strain and skin temperature; that in turn, improve thermal comfort and enhance endurance performance in the heat (Gagge et al., 1967; Lorenzo et al., 2010; Taylor, 2014).

Routine endurance training performed in temperate conditions, which elevates body temperature and initiates perfuse sweating, shares common adaptive responses to exercise-heat-acclimation such as; an earlier onset and an increase in sweating rate, a reduction in core temperature and a reduction in cardiovascular strain during exercise-heat-stress; which in turn, improves endurance performance in the heat (Piwonka et al., 1965; Strydom et al., 1966; Gisolfi and Robinson, 1969; Shvartz et al., 1977). As such, endurance trained individuals are considered to be partially heat acclimatized (Piwonka et al., 1965; Strydom et al., 1966; Gisolfi and Robinson, 1969). By the same token, its long been considered that endurance trained individuals have less adaptation potential and require fewer exercise-heat-exposures to achieve a plateau in heat acclimation responses, compared with untrained individuals (Pandolf et al., 1977; Shvartz et al., 1977). For example, following constant work rate heat acclimation, trained individuals acquired smaller thermal benefits during exercise-heat-stress than untrained individuals (Shvartz et al., 1977). Soldiers of the highest aerobic fitness required only four exercise-heat-acclimation exposures to achieve a plateau in the reduction of end-exercise rectal core temperature (*T*_re_); whereas, soldiers with the lowest aerobic fitness required eight exercise-heat-acclimation exposures (Pandolf et al., 1977). A limitation of these studies, reporting smaller and more rapid adaptations to heat acclimation in trained individuals, is that the observed plateau in heat acclimation adaptations may simply represent habituation to the constant exercise-heat-stress; resulting in a decline in the adaptation stimulus (Taylor, 2014). Recent studies that have maintained the endogenous thermal stimulus during controlled hyperthermia heat acclimation demonstrate comparable thermal and cardiovascular adaptations in endurance trained (Neal et al., 2016) and recreationally active individuals (Gibson et al., 2015).

Despite compelling evidence that exercise-heat-acclimation alleviates thermal strain and improves performance in the heat (Nielsen et al., 1997; Lorenzo et al., 2010), only 15% of athletes competing at the 2015 World Athletics Championships in the heat and humidity of Beijing heat acclimatized as part of their preparation (Periard et al., 2017). One possible explanation is that athletes consider their high level of fitness confers adaptations similar to heat acclimatization (Piwonka et al., 1965; Strydom et al., 1966; Gisolfi and Robinson, 1969); so they favor natural heat acclimatization in the few days preceding competition and prioritize other strategies to combat the heat such as fluid replacement and pre-cooling (Periard et al., 2017). Another explanation is that conventional exercise-heat-acclimation protocols can be costly, impractical and may interfere with an athlete’s training and taper: exercise-heat-acclimation typically involves access to an environmental chamber and precise control over exercising core temperature during endurance exercise. The completion of alternative heat acclimation methods, such as post-exercise sauna bathing (Scoon et al., 2007) and hot water immersion (HWI) (Zurawlew et al., 2016) have received increasing interest of late (Casadio et al., 2017). These methods are; accessible, time efficient, simple to administer and minimize disturbances to training and tapering. Recently, HWI after exercise in temperate conditions on 6 consecutive days initiated hallmarks of heat acclimation in recreationally active individuals (Zurawlew et al., 2016, 2018). Heat acclimation adaptations to post-exercise HWI included reductions in; *T*_re_ at rest, *T*_re_ at sweating onset and *T*_re_ during exercise-heat stress; in turn, restoring endurance performance in the heat to the level observed in temperate conditions (Zurawlew et al., 2016). Similar to controlled hyperthermia heat acclimation, post-exercise HWI ensures a maintenance of the daily thermal stimulus for adaptation (daily Δ*T*_re_; ≈2.1°C), since the termination of the HWI relies primarily on participants removing themselves due to thermal discomfort (Zurawlew et al., 2016, 2018).

It remains unknown whether heat acclimation adaptations to post-exercise HWI in a recreationally active population translate to an endurance trained population. As such, the aim of the current study was to compare the adaptation responses of endurance trained and recreationally active individuals following post-exercise HWI. We hypothesized that HWI after submaximal exercise in temperate conditions on 6 consecutive days would induce comparable heat acclimation adaptations in endurance trained and recreationally active individuals.

## Materials and Methods

### Participants

In accordance with previously defined classifications (De Pauw et al., 2013), eight endurance trained males (runners, *n* = 6 and triathletes, *n* = 2; age: 25 ± 4 years; body mass: 69 ± 4 kg; self-reported weekly endurance exercise: 9 ± 3 h; 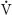
O_2max_: 68 ± 6 mL ⋅ kg^-1^ ⋅ min^-1^) and eight recreationally active males (age: 21 ± 3 years; body mass: 71 ± 9 kg; self-reported weekly endurance exercise: 3 ± 1 h; 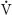
O_2max_: 54 ± 6 mL ⋅ kg^-1^ ⋅ min^-1^), participated in the study. All participants provided written informed consent to participate, were healthy, non-smokers, free from any known cardiovascular or metabolic diseases and were not taking any medication. Additionally, all participants had not been exposed to hot environmental conditions in the past 3 months and were not regular hot bath or sauna users. The study received local ethical approval and was conducted in accordance with the Declaration of Helsinki (2013).

### Study Design

A mixed-methods (between and within) repeated measures (PRE to POST) design was used to assess the effect of training status on heat acclimation adaptations. Endurance trained and recreationally active participants completed a 40-min submaximal treadmill run at 65% 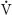
O_2max_ in the heat (33°C, 40% relative humidity; RH) before (PRE) and after (POST) heat acclimation, as described previously (Zurawlew et al., 2016). Heat acclimation involved a daily 40-min submaximal treadmill run at 65% 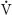
O_2max_ in temperate conditions (19°C), followed by a ≤40-min HWI (40°C water) on 6 consecutive days, as described previously (Zurawlew et al., 2016).

### Preliminary Measurements

In temperate conditions (19°C), a continuous incremental exercise test on a motorized treadmill (HP Cosmos Mercury 4.0, Nussdorf-Traunstein, Germany) assessed 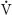
O_2max_, as previously described (Fortes et al., 2013). The interpolation of the running speed–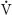
O_2_ relationship determined a running speed that elicited 65% 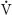
O_2max_. This speed was verified during steady state exercise with a 60-s expired gas sample collected by Douglas bag method, 30-min after the 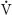
O_2max_ test. This individualized running speed was used during the submaximal exercise in both the experimental trials and the daily intervention.

### Experimental Trials

Participants were instructed to refrain from any exercise 24-h prior to, and on the day of experimental trials. In addition, participants were instructed to refrain from alcohol, caffeine or tobacco and to complete a diet diary 24-h prior to PRE. Twenty-four hours prior to POST, participants were instructed to replicate this food and fluid intake. On the morning of experimental trials, participants arrived at the laboratory fasted and were provided with a standardized breakfast (0.03 MJ kg^-1^) and a bolus of water equivalent to 7 mL ⋅ kg^-1^ of body mass. Following a 20-min seated rest in temperate conditions (19°C), dressed in a T-shirt, running shorts, socks and shoes, a venous blood sample was taken without stasis. A pre-exercise nude body mass was taken using a digital platform scale (Model 705; Seca, Hamburg, Germany) after voiding. A urine sample was provided and analyzed for urine specific gravity to confirm that participants were hydrated (<1.03) (Armstrong, 2005) using a handheld refractometer (Atago Uricon-Ne refractometer, NSG Precision cells, Farmingdale, NY, United States). If participants did not meet the hydration criteria they were provided with a 500-mL bolus of water and urine specific gravity was reanalyzed; exercise began only when urine specific gravity <1.03 (*n* = 1). Participants were instrumented for the exercise protocol, then rested in a temperate laboratory to establish baseline measures prior to beginning the exercise.

Dressed in running shorts, socks and shoes the participant entered the environmental chamber (33 ± 0°C, 40 ± 4% RH; Delta Environmental Systems, Chester, United Kingdom) and completed a submaximal treadmill run (40-min, 65% 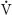
O_2max_, 1% gradient). *T*_re_, skin temperatures and heart rate (Polar FT1, Polar Electro, Kempele, Finland) were monitored continuously and local forearm sweat rate was measured every 20-s for the first 15-min of exercise. Physiological strain index (PhSI) was calculated, as previously described (Tikuisis et al., 2002). Expired gas samples (60-s) were collected by Douglas bag method to assess for 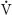
O_2_ and respiratory exchange ratio (RER) immediately prior to the 10th, 20th, 30th, and 40th min of exercise. Metabolic energy expenditure was calculated using 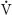
O_2_ and RER as described (Nishi, 1981). Rating of perceived exertion (RPE) (Borg, 1970) and thermal sensation (Hollies and Goldman, 1977) were recorded every 10-min of exercise. On completion of the exercise protocol, participants exited the environmental chamber and rested in temperate conditions, dressed in running shorts, socks, and shoes for 15-min. To estimate whole body sweat rate (WBSR), participants towel dried and provided a nude body mass following the seated rest. Participants were then provided with water equivalent to sweat losses and were free to leave the laboratory when *T*_re_ ≤ 38.5°C.

### Post-exercise Hot Water Immersion Intervention

Post-exercise HWI heat acclimation was completed on 6 consecutive days, as previously described (Zurawlew et al., 2016). During the intervention, participants were instructed to reduce their normal endurance exercise volume by that completed during the intervention in the laboratory and to consume their normal diet and fluid intake, including caffeine and alcohol (≤3 units per day). Participants arrived at the laboratory each day between 0600-h and 1000-h. A heart rate monitor and a rectal thermistor were fitted and the participant rested in temperate conditions (19°C) for 15-min. Following the seated rest, dressed in shorts, socks and trainers, in a hydrated state (urine specific gravity < 1.03), participants completed a 40-min submaximal run (65% 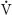
O_2max_, 1% gradient) on a motorized treadmill in temperate conditions (19°C). Within the first 20-min of exercise, participants consumed a bolus of water (5 mL ⋅ kg^-1^ of body mass). Following exercise, participants undertook a ≤40-min HWI (40°C), immersed to the neck dressed in shorts (2–3 min transition time). Immersion in hot water was terminated either at 40 min, when participants removed themselves due to thermal discomfort or when *T*_re_ exceeded the institutional ethical cut off (39.9°C). Following removal from the hot water, participants rested in temperate laboratory conditions, dressed in shorts for 15-min without fluids. Following which, participants towel dried and a nude body mass was recorded and adjusted for fluid intake as a measure of WBSR. Participants were free to leave the laboratory when *T*_re_ ≤ 38.5°C.

### Measurement and Instrumentation

#### Body Temperatures

*T*_re_ was measured using a flexible, sterile rectal thermistor (Henleys Medical Supplies Ltd., Herts, United Kingdom), self-inserted 10 cm beyond the rectal sphincter and recorded using a data logger (YSI model 4000 A, YSI, Dayton, OH, United States). An area under the curve (AUC) analysis was performed on *T*_re_ (time *T*_re_ was >38.5°C) during each post-exercise HWI exposure to assess for cumulative hyperthermia, as previously described (Cheuvront et al., 2008). Skin temperatures were measured during experimental trials using insulated thermistors (Grant EUS-U, Cambridge, United Kingdom) secured on the right side of the body at four locations: chest at a midpoint between the acromion process and the nipple; the lateral mid-bicep; the anterior mid-thigh; and, lateral calf and recorded using a data logger (Grant SQ2020, Cambridge, United Kingdom). Mean skin temperature (*T*_sk_) was calculated from the four sites using a weighted equation (Ramanathan, 1964).

#### Sweating Responses

Changes in dry nude body mass estimated WBSR during experimental trials and post-exercise HWI heat acclimation exposures. Dew point hygrometry measured local forearm sweat rate during the experimental trials, as previously described (Fortes et al., 2013). The individual relationships between local forearm sweat rate and *T*_re_ were used to calculate the onset of sweating (Cheuvront et al., 2009).

### Blood Sample Collection and Analysis

During experimental trials, prior to exercise a venous blood sample (6 mL) was collected into an EDTA vacutainer (BD, Oxford, United Kingdom) without stasis from an antecubital vein, following a 20-min seated rest to stabilize body fluids. Hemoglobin concentration (g ⋅ dL^-1^; Hemocue, Sheffield, United Kingdom) in duplicate and hematocrit (%) in triplicate (capillary tube method) were immediately assessed from aliquots of whole blood. The change in plasma volume was estimated by correcting the initial plasma volume at PRE for the percentage change in plasma volume at POST, as previously described (Dill and Costill, 1974).

### Statistical Analysis

A sample size calculation (G^∗^Power 3.1.2), using an alpha level of 0.05, power of 0.80 and a strong correlation of 0.7, was performed using data from a study comparing heat acclimation responses in endurance trained and untrained individuals (Shvartz et al., 1977). For a two-way (group × time) repeated measures ANOVA, a sample size of eight participants per group was calculated to detect a significant difference in the magnitude of reduction in end-exercise *T*_re_ (Δ0.3°C), between endurance trained and untrained individuals following heat acclimation. All data were checked for normality and sphericity and statistical significance was accepted at *P* < 0.05. Two-way repeated measures analysis of variance (ANOVA) with Greenhouse Geisser correction to the degrees of freedom (where necessary) were used to assess for main effects, i.e., differences between groups (endurance trained vs. recreationally active) and changes from PRE to POST during the experimental trials and from day 1 to day 6 of the intervention, as well as interaction effects (group × time). Bonferroni-adjusted pairwise comparisons were used where appropriate to determine where differences occurred. Independent *t*-tests assessed for differences in total HWI time and total AUC between endurance trained and recreationally active. The magnitude of effect was reported using Cohen’s *d*, where 0.2, 0.5, and 0.8 represent small, medium and large effects, respectively (Cohen, 1988). Pearson’s correlations were used to determine the strength of the relationship between training status or aerobic fitness (habitual weekly endurance exercise and 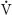
O_2max_) and the reduction in end-exercise *T*_re_ and heart rate, and between the thermal stimulus (total AUC) during the heat acclimation intervention and the reduction in end-exercise *T*_re_ and heart rate. Data are presented as mean ± standard deviation (SD) and were analyzed using SPSS version 24 (IBM Corporation, Armonk, NY, United States), or GraphPad Prism Version 5.02 (GraphPad Software Inc., La Jolla, CA, United States).

## Results

### Intervention

All participants completed a 40-min treadmill run at 65% 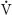
O_2max_ followed by HWI (≤40 min) on 6 consecutive days. Compared to their typical endurance exercise volume, during the intervention, weekly endurance exercise volume was unchanged for endurance trained (-1 ± 2 h; *P* > 0.05) and increased in recreationally active individuals (+2 ± 0 h; *P* < 0.01). During the 6-day intervention, HWI duration increased from day 1 to day 6 (*P* < 0.05); ensuring a maintenance of the endogenous stimulus for adaptation, with a similar AUC and end-HWI *T*_re_ between day 1 and day 6 (*P* > 0.05; Table 1). In addition, total immersion time (*P* = 0.08, *d* = 1.0) and total AUC (*P* = 0.08, *d* = 0.8) during the 6-day intervention tended to be greater in endurance trained than recreationally active individuals (Table 1).

**Table 1:** The influence of 40-min submaximal running at 65% 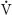
O_2max_ in temperate conditions followed by post-exercise hot water immersion in 40°C water on thermoregulatory variables, heart rate and immersion time in endurance trained and recreationally active participants.

	Endurance trained		Recreationally active	
	Day 1	Day 6	Day 1	Day 6
**Submaximal exercise**				
End-exercise *T*_re_ (°C)	38.37 ± 0.48	38.27 ± 0.43	38.34 ± 0.32	38.22 ± 0.23
End-exercise heart rate (beats min^-1^)^∗^	147 ± 13	144 ± 10	150 ± 9	144 ± 9
**Hot water immersion**				
End-immersion *T*_re_ (°C)	39.44 ± 0.44	39.36 ± 0.31	39.15 ± 0.18	39.21 ± 0.20
Immersion time (min)^∗∗^	35 ± 8	40 ± 0	28 ± 5	40 ± 1
*n* completing 40-min immersion	5 of 8	8 of 8	0 of 8	7 of 8
**Submaximal exercise and hot water immersion**				
WBSR (L ⋅ h^-1^)^∗^^#^	1.08 ± 0.34	1.25 ± 0.26	0.72 ± 0.17	0.95 ± 0.18
AUC (°C ⋅ min^-1^)	33 ± 24	29 ± 15	18 ± 7	20 ± 7

*T_re_, rectal temperature; AUC, area under the curve. ^∗^P < 0.05 and ^∗∗^P < 0.01, main effect of time. ^#^P < 0.05, main effect of group. Data displayed as mean ± SD.*

### Experimental Trials

**FIGURE 1:**
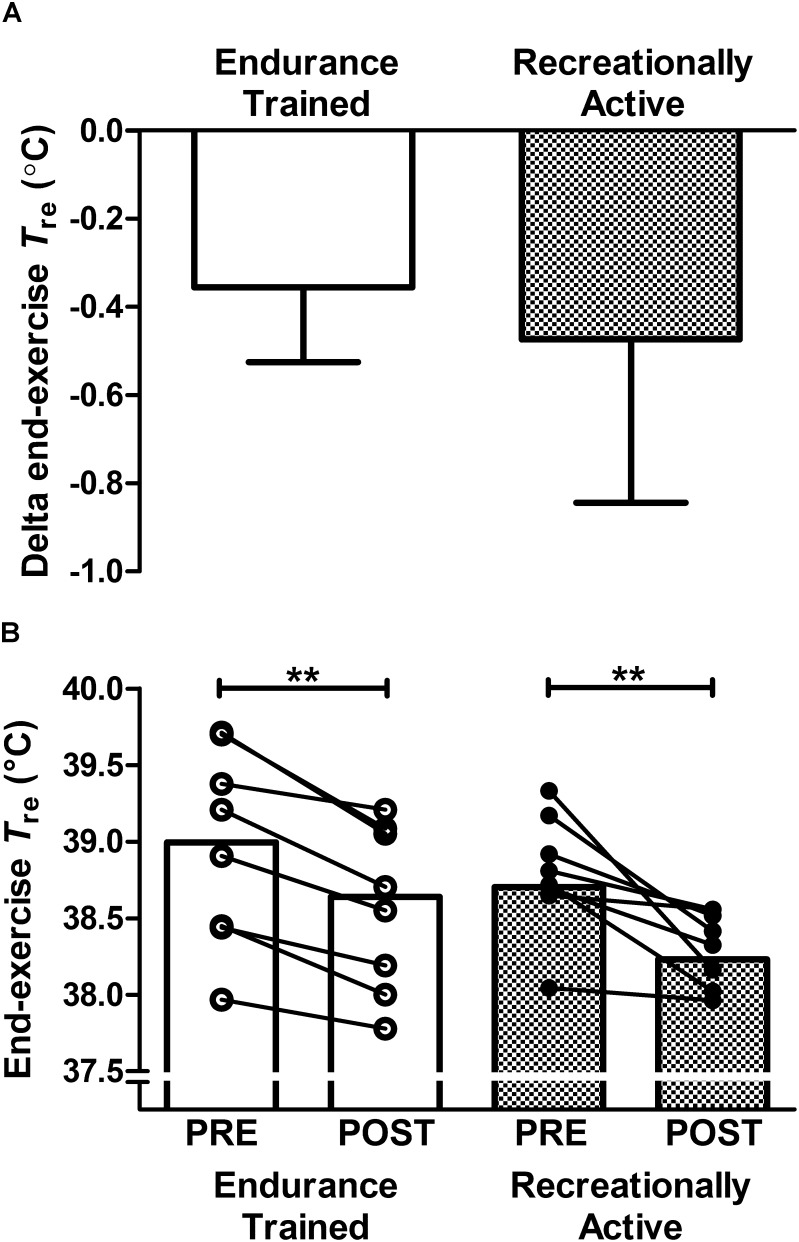
Effect of 6-day post-exercise hot water immersion heat acclimation on end-exercise rectal core temperature (*T*_re_) following a 40-min submaximal treadmill run at 65% 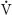
O_2max_ in the heat (33°C, 40% RH) in endurance trained and recreationally active individuals. Bars represent mean ± SD of the PRE to POST change **(A)** and mean at PRE and POST **(B)** for end-exercise *T*_re_. Lines between bars represent individual participant responses. ^∗∗^*P* < 0.01 denotes POST lower than PRE (main effect of time).

No significant interaction effects (group × time; *P* > 0.05) demonstrate that training status did not influence the observed adaptations to the 6-day post-exercise HWI intervention, for measures of: end-exercise *T*_re_ (Figure 1A); resting *T*_re_; *T*_re_ at sweating onset; Δ*T*_re_ during exercise; end-exercise *T*_sk_; end-exercise *T*_re_–*T*_sk_ gradient; end-exercise PhSI; end-exercise RPE, end-exercise thermal sensation, mean 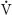
O_2_, mean RER or mean metabolic energy expenditure (Table 2). Training status did not relate strongly to the magnitude of thermal adaptation; since the reduction in end-exercise *T*_re_ during exercise-heat-stress was not strongly correlated with either habitual endurance exercise volume (*r* = 0.35, *P* > 0.05) or 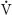
O_2max_ (*r* = 0.29, *P* > 0.05). In endurance trained individuals, a larger thermal stimulus during the HWI intervention (total AUC 63–320°C min^-1^) was strongly associated with a larger reduction in end-exercise *T*_re_ (*r* = -0.71; *P* < 0.05). Moreover, post-exercise HWI reduced thermal strain during exercise-heat stress in all 16 participants, supported by a main effect of time (PRE vs. POST) for end-exercise *T*_re_ (PRE; 38.85 ± 0.49°C, POST; 38.43 ± 0.42°C, *P* < 0.01, *d* = 0.9; Figure 1B); albeit, one recreationally active participant experienced only a 0.08°C reduction in end-exercise *T*_re_. Contrary to the notion that the most highly trained would benefit the least from the HWI intervention, the most accomplished endurance trained participant, an international marathon runner (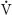
O_2max_: 81 mL ⋅ kg^-1^ ⋅ min^-1^ and road half-marathon PB: 66 min) experienced a meaningful reduction in end-exercise *T*_re_ during exercise-heat-stress (PRE; 38.94°C, POST; 38.62°C).

**Table 2:** Effect of 6-day post-exercise hot water immersion heat acclimation on thermal, cardiovascular, metabolic, and perceptual responses at rest and to 40-min submaximal treadmill running at 65% 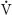
O_2max_ in the heat (33°C, 40% RH) in endurance trained and recreationally active participants.

	Endurance trained	Recreationally active
*T*_re_ at sweating onset (°C)^∗∗^	-0.22 ± 0.24	-0.23 ± 0.29
Δ *T*_re_ during exercise (°C)^∗^^##^	-0.19 ± 0.35	-0.25 ± 0.27
End-exercise *T*_sk_ (°C)^∗∗^	-0.67 ± 0.38	-0.75 ± 0.70
End-exercise *T*_re_–*T*_sk_ gradient (°C)^∗^	0.31 ± 0.42	0.27 ± 0.62
WBSR (L ⋅ h^-1^)^##^	0.13 ± 0.02	-0.03 ± 0.25
End-exercise heart rate (beats ⋅ min^-1^)^∗∗^	-4 ± 5	-15 ± 7 ^††^
End-exercise PhSI (0–10)^∗∗^^#^	-1 ± 1	-1 ± 1
Mean 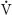 O_2_ (L ⋅ min^-1^)^∗∗^^##^	-0.1 ± 0.1	-0.1 ± 0.2
Mean metabolic energy expenditure (W)^∗∗^^##^	-28 ± 31	-40 ± 50
End-exercise RPE (6–20)^∗^	-1 ± 1	-2 ± 3
End-exercise thermal sensation (1–13)^∗∗^	-1 ± 1	-1 ± 1

*T_re_, rectal temperature; T_sk_, mean skin temperature; WBSR, whole body sweat rate; PhSI, physiological strain index; RPE, rating of perceived exertion. Data displayed as mean ± SD of the PRE to POST change. ^∗^P < 0.05 and ^∗∗^P < 0.01, denotes POST different than PRE (main effect of time). ^#^P < 0.05 and ^##^P < 0.01, denotes endurance trained different than recreationally active (main effect of group).*

*^††^P < 0.01, denotes POST lower than PRE within group (post hoc time effect).*

**FIGURE 2:**
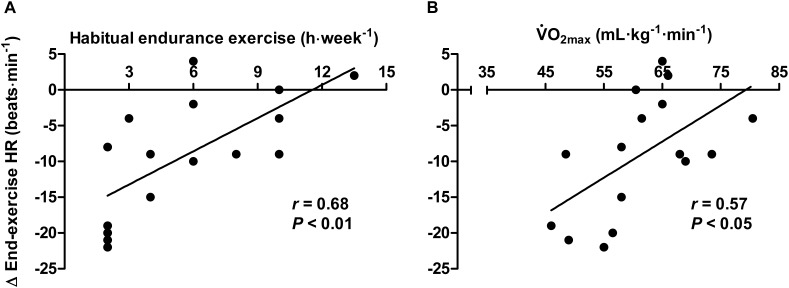
Relationship between habitual endurance exercise volume **(A)** and aerobic fitness **(B)** and the change in heart rate response to exercise-heat-stress after 6-day post-exercise hot water immersion heat acclimation.

Recreationally active individuals experienced a smaller thermal stimulus during the HWI intervention (total AUC 58–197°C ⋅ min^-1^) than endurance trained individuals, since they terminated HWI sooner due to thermal discomfort (Table 1). The thermal stimulus during the post-exercise HWI intervention was not strongly related to the reduction in end-exercise *T*_re_ in recreationally active individuals (*r* = 0.12, *P* > 0.05). As such, there appear to be other drivers contributing to the observed adaptations in recreationally active individuals, beyond the total AUC. An interaction effect (group × time; *P* < 0.01) was observed for end-exercise heart rate, with a significant reduction from PRE to POST in recreationally active (PRE; 178 ± 12 beats ⋅ min^-1^, POST; 163 ± 9 beats ⋅ min^-1^, *P* < 0.01, *d* = 1.4), but not endurance trained individuals (PRE; 167 ± 15 beats ⋅ min^-1^, POST; 163 ± 16 beats ⋅ min^-1^, *P* > 0.05, *d* = 0.2; Table 2). Correlations suggest that the decrease in end-exercise heart rate during exercise-heat-stress after the HWI intervention was relatively strongly related to habitual exercise volume (*r* = 0.68, *P* < 0.01; Figure 2A) and aerobic fitness (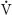
O_2max_; *r* = 0.57, *P* < 0.05; Figure 2B); whereby, those with a higher habitual exercise volume and aerobic fitness demonstrated a smaller reduction in end-exercise heart rate after the 6-day post-exercise HWI intervention. In contrast, the thermal stimulus during the HWI intervention (total AUC) was not strongly related to the decrease in end-exercise heart rate during exercise-heat-stress (*r* = 0.14, *P* > 0.05).

Other hallmark heat acclimation adaptations were achieved following post-exercise HWI (main effect of time, PRE vs. POST, *n* = 16), including reductions in: resting *T*_re_ (PRE; 36.91 ± 0.31°C, POST; 36.71 ± 0.35°C, *P* < 0.01, *d* = 0.6); *T*_re_ at sweating onset (*P* < 0.01, *d* = 0.6); Δ*T*_re_ during exercise (*P* < 0.05, *d* = 0.5); end-exercise heart rate (*P* < 0.01, *d* = 0.7); *T*_sk_ (*P* < 0.01, *d* = 0.8); *T*_re_–*T*_sk_ gradient (*P* < 0.05, *d* = 0.3); PhSI (*P* < 0.01, *d* = 0.9); RPE (“fairly hard” to “fairly light,” *P* < 0.05, *d* = 0.7); thermal sensation (“hot” to “uncomfortably warm,” *P* < 0.01, *d* = 1.0); mean 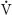
O_2_ (*P* < 0.01, *d* = 0.2) and mean energy expenditure (PRE; 1037 ± 160 W, POST; 1003 ± 160 W, *P* < 0.01, *d* = 0.2; Table 2). No main effect for time (PRE vs. POST; *n* = 16) was observed for WBSR (1.02 ± 0.35 L ⋅ h^-1^ to 1.07 ± 0.33 L ⋅ h^-1^, *P* > 0.05, Table 2) or mean RER (*P* > 0.05) and the relative changes in plasma volume were not significantly different in endurance trained (4 ± 8%) or recreationally active participants (3 ± 7%; *P* > 0.05, *d* = 0.6). There was no main effect for training status (endurance trained vs. recreationally active), for measures of resting *T*_re_, *T*_re_ at sweating onset, mean exercising RER and measures taken at end-exercise including: *T*_re_; heart rate; *T*_sk_; *T*_re_–*T*_sk_ gradient; RPE and thermal sensation (*P* > 0.05). However, Δ*T*_re_ during exercise, end-exercise PhSI, WBSR, mean 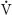
O_2_ and mean metabolic energy expenditure were greater in endurance trained compared with recreationally active individuals (*P* < 0.05).

## Discussion

The present study sought to compare heat acclimation adaptations in endurance trained and recreationally active individuals after a 6-day post-exercise HWI intervention. In agreement with our hypothesis, the new and noteworthy finding is that HWI brought about comparable heat acclimation adaptations in endurance trained and recreationally active individuals. Hallmark heat acclimation adaptations, observed in endurance trained and recreationally active individuals, included reductions in resting *T*_re_ and reductions in: end-exercise *T*_re_; end-exercise PhSI; *T*_re_ at sweating onset and *T*_sk_ during exercise-heat-stress. Furthermore, training status did not significantly influence observed reductions in thermal sensation or RPE during exercise-heat-stress after the HWI intervention. The observed benefits were achieved by exposure to a large thermal stimulus for adaptation during the daily heat acclimation sessions (change in *T*_re_≈2°C; *T*_sk_ = 40°C); despite no significant changes in WBSR or plasma volume.

The heat acclimation benefit of the HWI intervention for endurance trained individuals is emphasized by the association between the thermal stimulus (total AUC °C ⋅ min^-1^) and the reduction in end-exercise *T*_re_ during exercise-heat-stress (*r* = -0.71); whereby, thermal strain during exercise-heat-stress was reduced most in endurance trained individuals who experienced the greatest thermal stimulus during the HWI intervention. Contrary to the notion that the most highly trained individuals would benefit the least (Pandolf et al., 1977; Shvartz et al., 1977), our most accomplished endurance performer, an international marathon runner, experienced a meaningful reduction in end-exercise *T*_re_ after the HWI intervention (0.32°C). Nevertheless, endurance trained individuals tended to require a greater thermal stimulus during the HWI intervention to achieve a similar reduction in thermal strain as recreationally active individuals. This was likely a consequence of the endurance trained individuals’ partial heat acclimatization status and associated increased heat tolerance (Piwonka et al., 1965; Strydom et al., 1966; Gisolfi and Robinson, 1969; Selkirk and McLellan, 2001). The responsible mechanism(s) for the reduction in resting *T*_re_ with post-exercise HWI (Zurawlew et al., 2016) and exercise-heat-acclimation (Tyler et al., 2016) require elucidation. Reductions in resting *T*_re_ have been related to a lowering of metabolic rate in seasonal heat acclimatization (Buguet et al., 1988) and to endurance training adaptations (Baum et al., 1976). The coupling of the reduction in resting *T*_re_ (-0.20°C) and *T*_re_ at sweating onset (-0.22°C) likely accounts for the further reduction in thermal strain during exercise heat stress after the HWI intervention (end exercise *T*_re_ -0.42°C): heat loss via sweating and cutaneous vasodilation are initiated at lower thermoregulatory thresholds after heat acclimation (Buono et al., 1998).

As a consequence of their habitual exercise training, endurance trained individuals are considered to be further along the heat adaptation continuum than recreationally active individuals; reducing their adaptation potential (Taylor, 2014). It’s perhaps not surprising then, and in keeping with exercise-heat-acclimation findings (Shvartz et al., 1977), that a greater reduction in exercising heart rate was observed in recreationally active than endurance trained individuals after the HWI intervention (-15 vs. -4 beats ⋅ min^-1^ in endurance trained). Indeed, the magnitude of the reduction in heart rate was associated with habitual endurance exercise volume (*r* = 0.68) and aerobic fitness (*r* = 0.57); whereby, heart rate was reduced most in those completing less habitual endurance exercise and those with lower aerobic fitness (Figure 2). Likely, the passive heat stimulus during HWI elicited the notable reduction in cardiovascular strain in recreationally active individuals in the present study; in agreement with the findings of others (Brebner et al., 1961; Brazaitis and Skurvydas, 2010). It’s unlikely that the reduction in heart rate was due to the daily exercise as we have previously shown no improvements in cardiovascular fitness (i.e., no reduction in heart rate or 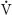
O_2_) in recreationally active individuals who performed the same daily exercise intervention followed by a thermoneutral bath (Zurawlew et al., 2016). The more notable reduction in heart rate in recreationally active individuals after the HWI intervention likely relates to alterations in cardiac autonomic regulation (Periard et al., 2016); adaptations already possessed by the endurance trained individuals (Carter et al., 2003). We show no obvious plasma volume expansion and a similar widening of the *T*_re_–*T*_sk_ gradient in recreationally active and endurance trained after the intervention; both mechanisms are often posited to reduce cardiovascular strain with heat acclimation (Periard et al., 2015).

Hallmark adaptations to the heat have long been considered to include an expansion in resting plasma volume (Greenleaf et al., 1983) and an increase in WBSR during exercise-heat-stress (Wyndham and Strydom, 1969). Corroborating our recent work in recreationally active individuals (Zurawlew et al., 2016, 2018), the current findings demonstrate that post-exercise HWI also reduces thermal strain during exercise-heat-stress in endurance trained individuals; despite no obvious increase in plasma volume or WBSR. It’s noteworthy that a recent meta-analysis highlighted the rather modest and variable plasma volume expansion (+4 ± 5%) and increase in WBSR (+5 ± 11%) in short-term heat acclimation studies (<7 exposures) (Tyler et al., 2016). Typically, >7 exercise-heat-acclimation exposures are required to initiate an increase in WBSR; but even then, these responses are highly variable (+29 ± 29%) (Tyler et al., 2016). The semi-recumbent body position and hydrostatic forces during HWI may maintain central vascular volume and in-turn reduce fluid regulatory stress and the stimulus for plasma volume expansion (Nagashima et al., 1999; Bradford et al., 2015). However, the absence of an expansion in plasma volume may be associated with errors in estimating relative changes in plasma volume using hemoglobin and hematocrit; therefore, future research should verify this finding using tracer techniques. Immersing the skin in hot water has been shown to reduce sweat gland activity and the stimulus for an increase in sweating (Hertig et al., 1961; Brebner and Kerslake, 1968). However, a more likely explanation for the lack of an increase in WBSR with HWI is that the decrease in *T*_re_ at sweating onset (-0.22°C) was offset by the decrease in resting *T*_re_ (-0.20°C). No increase in WBSR during exercise-heat-stress after the HWI intervention may provide additional thermoregulatory and performance benefits during exercise-heat-stress, by constraining dehydration and preserving central blood volume (Montain and Coyle, 1992). Heat acclimation by post-exercise HWI may limit the “wasteful overproduction of sweat” (Mitchell et al., 1976); particularly important in high humidity conditions when evaporative heat loss is limited and when sweat may drip from the skin. Notwithstanding, we recognize that the relatively modest exercise-heat stress for experimental trials (65% 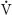
O_2max_, 33°C, 40% RH) may have masked an increase in WBSR (Poirier et al., 2015); as such, studies should investigate the influence of heat acclimation by post-exercise HWI on WBSR during a more uncompensable exercise scenario.

Despite evidence that exercise-heat-acclimation alleviates thermal strain and improves performance in the heat (Nielsen et al., 1997; Lorenzo et al., 2010), practical barriers limit athlete engagement with current exercise-heat-acclimation recommendations (Tyler et al., 2016; Casadio et al., 2017; Periard et al., 2017). As such, there has been an increasing interest of late in practical heat acclimation methods; including, training in temperate conditions whilst wearing additional clothing (Stevens et al., 2018) and post-exercise HWI (Zurawlew et al., 2016, 2018). Exercise in temperate conditions wearing additional clothing may provide the necessary elevations in core and skin temperature for adaptation (Dawson et al., 1989; Ely et al., 2018). However, a recent field study in triathletes showed that training in temperate conditions (18°C) wearing additional clothing was not an effective heat acclimation strategy (Stevens et al., 2018). For endurance trained athletes residing and training in temperate conditions, the current findings support the recommendation that incorporating a hot bath (lasting up to 40 min) in the post-exercise washing routine represents an effective and accessible heat acclimation strategy to prepare for competition in the heat. Taking a hot bath after temperate exercise limits interference with an athlete’s training and taper and does not require access to an environmental chamber or precise control over exercising core temperature. The current findings (delta end exercise *T*_re_ -0.42°C), and our previous work (Zurawlew et al., 2016, 2018), show that the magnitude of adaptations following post-exercise HWI compare favorably with exercise-heat-acclimation interventions, as reported in a recent meta-analysis (delta exercise core temperature -0.34°C) (Tyler et al., 2016). Therefore, the findings from the current study, considered alongside the extant literature, do not support the notion that exercise-heat-acclimation evokes superior adaptation and should be recommended in favor of passive heat acclimation (Periard et al., 2016; Tyler et al., 2016). Notwithstanding, future studies should directly compare post-exercise HWI and exercise-heat-acclimation and confirm whether the observed adaptations in endurance trained individuals translate to improved aerobic performance; as was previously observed in recreationally active individuals (Zurawlew et al., 2016). A small handful of studies provide evidence of heat acclimation with repeated HWI alone (without prior exercise) (Brebner et al., 1961; Bonner et al., 1976; Brazaitis and Skurvydas, 2010); as such, studies may wish to compare the efficacy of HWI with and without prior exercise. However, unpublished observations in our laboratory show a larger thermal stimulus (daily AUC °C ⋅ min^-1^) for post-exercise HWI than for the HWI alone strategies used in two of these studies (Brebner et al., 1961; Bonner et al., 1976). Moreover, we have concerns regarding participant safety and tolerance to the unpleasantly high water temperature (44°C) used in another of these studies (Brazaitis and Skurvydas, 2010). On the one hand, studies should determine whether meaningful heat acclimation can be achieved by fewer and/or shorter post-exercise HWI exposures. On the other hand, mindful of safety and practical constraints, exploring whether the observed adaptations can be further augmented by increasing the intensity of the prior exercise, or increasing the number and/or duration of HWI exposures requires investigation. Finally, studies should also determine the rate of decay of heat acclimation after the 6-day post-exercise HWI intervention and whether the adaptations observed herein translate to females.

## Conclusion

Hot water immersion after exercise in temperate conditions on 6 consecutive days reduced thermal strain during exercise-heat-stress in endurance trained and recreationally active individuals. For high level athletes residing and training in temperate conditions, incorporating a hot bath in the post-exercise washing routine represents an effective heat acclimation strategy to prepare for major competition in the heat.

## Author Contributions

NW had primary responsibility for the final content. NW, JM, and MZ were involved in the conception of the project and development of the research plan. MZ led the data collection. NW, JM, and MZ performed the data analysis, interpreted the data and prepared the manuscript.

## Conflict of Interest Statement

The authors declare that the research was conducted in the absence of any commercial or financial relationships that could be construed as a potential conflict of interest.
